# What are the vector species of the Oropouche virus?

**DOI:** 10.1002/ps.70460

**Published:** 2025-12-24

**Authors:** Constância Flávia Junqueira Ayres, Bruna Lais Sena do Nascimento, Daniele Barbosa de Almeida Medeiros, Joaquim Pinto Nunes Neto

**Affiliations:** ^1^ Department of Entomology Aggeu Magalhães Institute (IAM), Oswaldo Cruz Foundation (Fiocruz/PE), Campus da UFPE Recife Brazil; ^2^ Department of Arbovirology and Hemorrhagic Fevers Ananindeua PA Brazil

**Keywords:** Oropouche virus, vectors, *Culicoides paraensis*, *Culex*, vector competence, vector surveillance

## Abstract

Oropouche fever, caused by the arbovirus Oropouche virus (OROV; Peribunyaviridae: *Orthobunyavirus:Orthobunyavirus oropouche*), has significantly increased in Brazil, with >25 000 confirmed cases during the 2023–2025 outbreak. Historically endemic to the Amazon region, OROV is now spreading beyond its traditional range. The vectorial dynamics of OROV transmission remain under scrutiny. Although *Culicoides paraensis* is identified as the primary urban vector, the role of *Culex quinquefasciatus* and other culicids is still under debate, as laboratory studies indicate limited vector competence. Entomological investigations highlight the challenges of detecting OROV in nature, with low virus isolation rates from biting midges and mosquitoes. Vertical transmission in mosquitoes suggests potential alternative mechanisms for virus maintenance. Recent outbreaks in the Extra‐Amazonian areas emphasize the importance of comprehensive vector studies to refine control strategies. This review synthesizes seven decades of research on OROV epidemiology and vector competence, identifying critical knowledge gaps, such as the definitive wild cycle vector, and we suggest a research agenda. Addressing these gaps is pivotal for preventing further geographical spread, reducing fatalities, and mitigating the public health impact of this emerging arbovirus. © 2025 The Author(s). *Pest Management Science* published by John Wiley & Sons Ltd on behalf of Society of Chemical Industry.

## Graphical abstract

Here, we review the timeline of Oropouche virus (OROV) detection in various hematophagous Diptera, from 1955 to date, including mosquitoes and midges. All vector competence experiments also are described. The results suggest that *Culicoides* are the primary vectors.

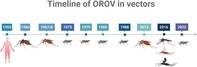

## 1 INTRODUCTION

Brazil has been experiencing a considerable increase in the number of cases of Oropouche fever, a self‐limited febrile illness caused by an arbovirus, named Oropouche virus (OROV)–*Orthobunyavirus oropoucheense*, a member of the family Peribunyaviridae.^1^

**Figure 1:**
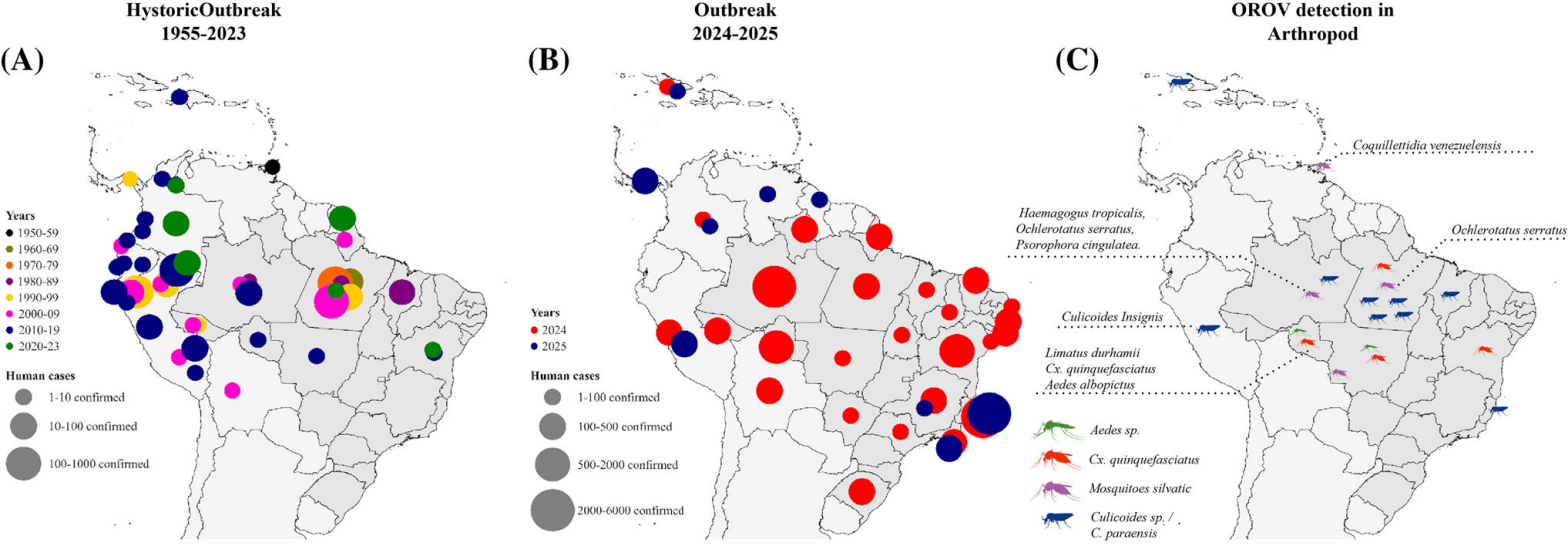
Maps showing: (A) the historical distribution of Oropouche fever epidemics between 1955 and 2023; (B) the distribution of confirmed OROV cases reported in 2024 and during the first half of 2025; and (C) the geographical distribution of arthropod species testing positive for OROV.

The virus is endemic to the Amazon region and has been known since 1955, when it was first isolated in Trinidad & Tobago.^2^ More than 30 epidemics have occurred in the Amazon basin, especially in Brazil, and it is estimated that >500 000 people have been infected already, in urban centers as well as peri‐urban or rural areas^3^, ^4^ (Fig. 1).

However, in this latest epidemic, cases are occurring outside the virus's endemic region.^5^ In 2024 alone, >13 000 cases have been confirmed in Brazil.^6^ Additionally, countries in Latin America, including Bolivia, Colombia, Cuba, the Dominican Republic and Peru, have reported OROV outbreaks, highlighting the rapid spread of this arbovirus.^7^

Besides the severe clinical signs in cases reported previously, such as meningitis, encephalitis and meningoencephalitis,^8^, ^9^, ^10^ the 2023–2025 OROV outbreak has recorded the first documented cases of Guillain–Barré syndrome,^11^ fatal outcomes,^12^ abortion, stillbirth^13^, ^14^, ^15^ and microcephaly^16^ associated with OROV infections.

Outside the Amazon rainforest, OROV cases have been concentrated in peri‐urban and rural areas, often linked to cocoa, sugarcane and banana cultivation. It is worth noting that in the Amazon region, major urban centers, such as Belém and Manaus, are located near wildland interfaces.^17^

In most published studies, *Culicoides paraensis* is described as the main vector in the urban cycle, whereas *Culex quinquefasciatus* is considered an occasional secondary vector.^18^ However, it is important to understand how this knowledge was constructed and whether this can be adopted as true in the 2023–2025 OROV outbreak. This information is relevant for estimating the risk of population exposure, predicting future outbreaks and developing effective control strategies to prevent the spread and increase in cases of the disease.

The objective of this review is to systematize the knowledge that has been built over the last seven decades, since the virus was first isolated, to understand the process of incriminating possible vectors in transmission, and to outline future priorities, including critical knowledge gaps that must be addressed to prevent the rapid spread of the virus to other regions of the world, and to reduce the risk of further fatalities.

**Figure 2:**
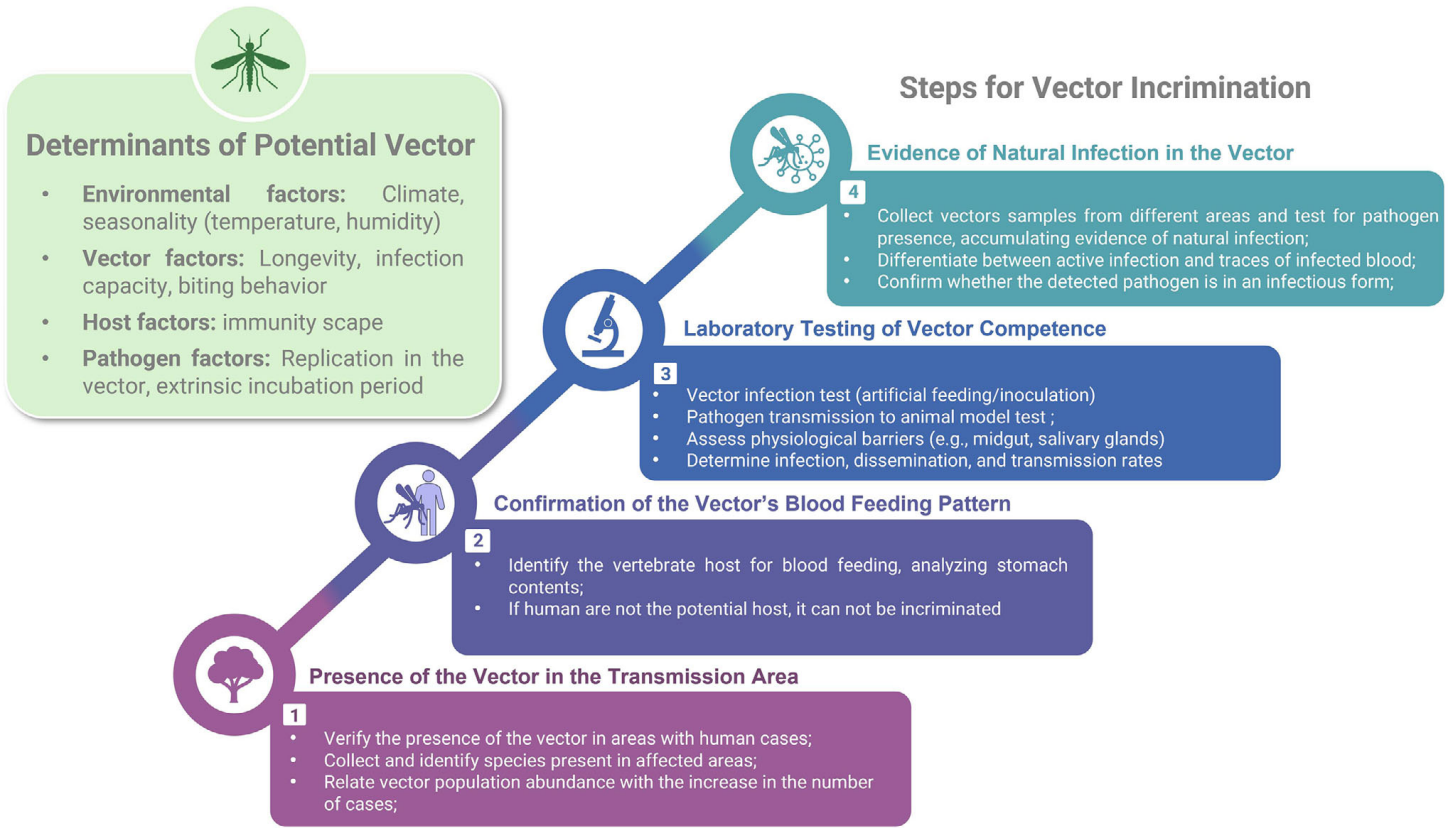
Step‐by‐step diagram for incriminating vector species.

According to Eldridge (2000),^19^ several steps are necessary to suggest that a given species is a vector of a pathogen. Some clues can be followed to begin an investigation into the incrimination of a vector (Fig. 2). The first, and most obvious, is to verify the presence of the species in the place where the transmission is occurring. If the number of cases of the disease is quite high, it is expected to observe the presence of this species in high abundance in the environment, which would justify the sudden increase in the number of cases. The transmission peak of some diseases occurs precisely as a result of the increase in vector population density, which may be associated with several abiotic factors or even the natural life cycle of the suspected vector species. In such cases, when outbreaks or epidemics of a given pathogen occur in a certain area, even if there is no structure to detect the pathogen in vector samples, it is of great importance to characterize the invertebrate fauna that could be carrying that pathogen. This initial characterization will guide the next steps for future investigations. Secondly, we need to be sure that the species in question commonly feeds on the vertebrate host. If this does not occur, even if it is abundant in the environment during an outbreak of the disease, it should not be evaluated as the main suspect. Thirdly, once the most common species during disease transmission have been identified, it is necessary to demonstrate that the species can be infected by and transmit the pathogen. This must be tested in a laboratory with biosafety conditions. These experiments investigate the vector competence of the suspected vector—an innate characteristic—by evaluating the potential barriers that may limit its ability to transmit the pathogen. Ideally, vector competence experiments should be carried out using the same parasite load observed in the vertebrate host to simulate natural conditions. Experiments using a pathogen load significantly higher than what is naturally found in the host may lead to increased replication of the pathogen in the vector, which might not reflect real‐world transmission dynamics.

Lastly, it is important to accumulate data from records of natural infection by the pathogen in the particular species, preferably in different locations. It is important to highlight that this natural infection must involve the infectious form of the pathogen, as the mere presence of the pathogen in samples from the species under investigation does not necessarily mean that it can be transmitted. Infection may result from recent feeding on an infected host, but it does not guarantee that the pathogen will replicate, complete the extrinsic incubation period, and reach the salivary glands to be passed to a new host.

By completing these four steps, it is possible to incriminate an invertebrate species as the main vector in the transmission cycle of a given disease‐causing pathogen.

Considering these criteria, and returning to the Oropouche fever case, a we reviewed what has been done to incriminate the currently known vectors, keeping in mind that there are two transmission cycles: the sylvatic and the urban environment cylce.

## 2 SEARCH STRATEGY AND SELECTION CRITERIA

**Figure 3:**
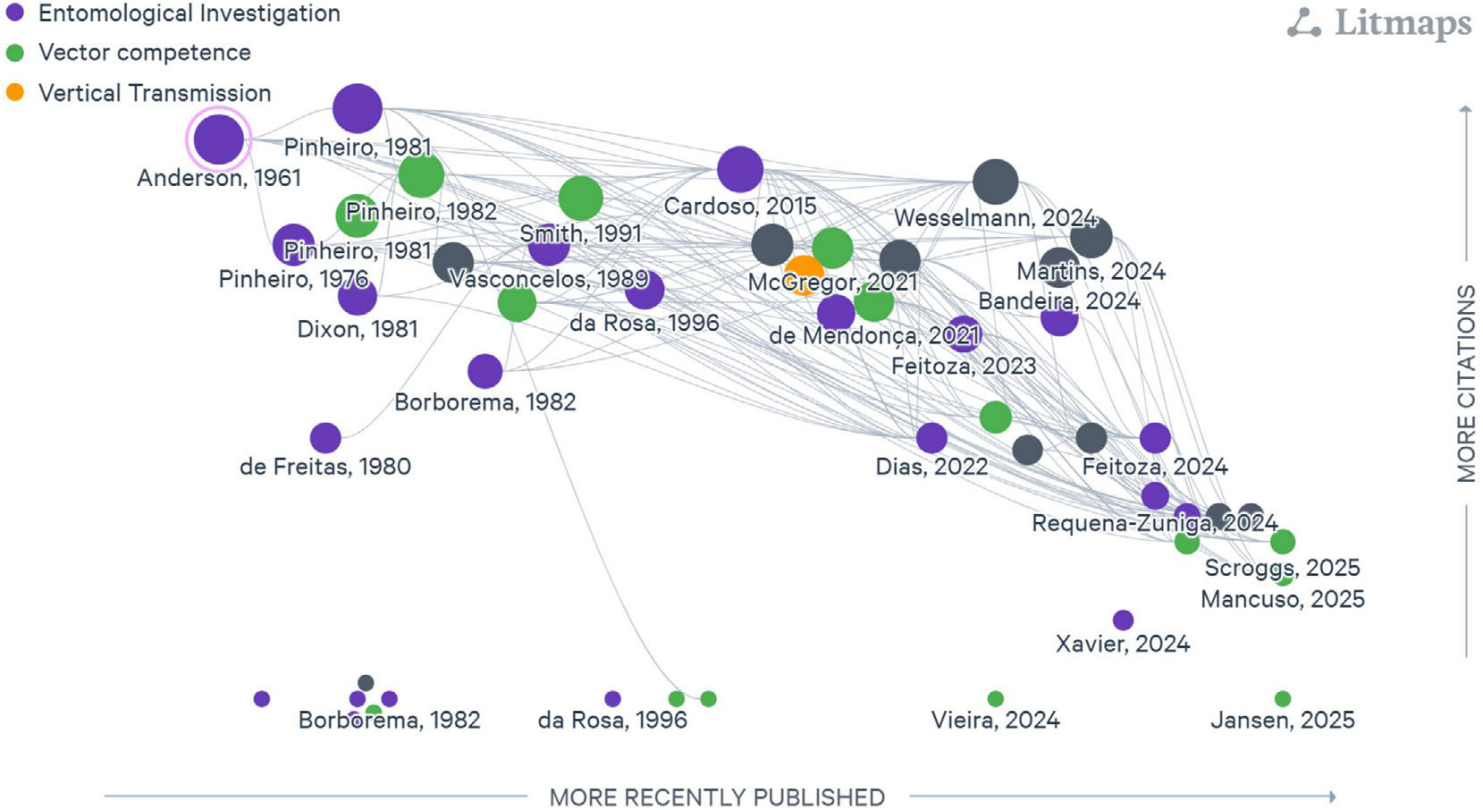
Literature on Oropouche virus vectors. Network visualization of scientific publications related to OROV vectors, classified by study type: entomological surveillance (purple), vector competence (green) and vertical transmission (orange). The gray dots are articles related to the disease and not necessarily to the study of vectors. Dots represent the articles used here, and lines are the connections among them. The dot size represents the citation frequency, and the position along the horizontal axis indicates year of publication, with more recent studies on the right.

This review includes references obtained through searches of PubMed, institutional libraries and internal collections, covering the period from 1955, when the OROV was first isolated in Trinidad and Tobago, to the present (August 2025), using the search term ‘Oropouche.’ All articles referring to vector research were included. Preprint articles and unpublished data, such as conference presentations, personal notes from research notebooks of leading researchers, and webinars, also were considered. Review articles that did not present new data were not considered. Additionally, we used LitMap^20^ to identify connections among publications, to highlight those that were most cited and the areas that need further studies (Fig. 3).

When citing the mosquito species, we preserved the original taxonomic nomenclature as presented in the primary sources. However, recent taxonomic revisions were included in parentheses, where applicable, to reflect updated classifications based on contemporary entomological standards. These updates follow the nomenclatural rules established by the International Code of Zoological Nomenclature (ICZN) and are informed by current authoritative databases, such as the Mosquito Taxonomic Inventory (MTI) and Systema Dipterorum. This approach ensures both historical consistency and scientific accuracy in line with modern taxonomy.

## 3 HISTORICAL OF VECTOR INVESTIGATIONS OF OROPOUCHE VIRUS

**Figure 4:**
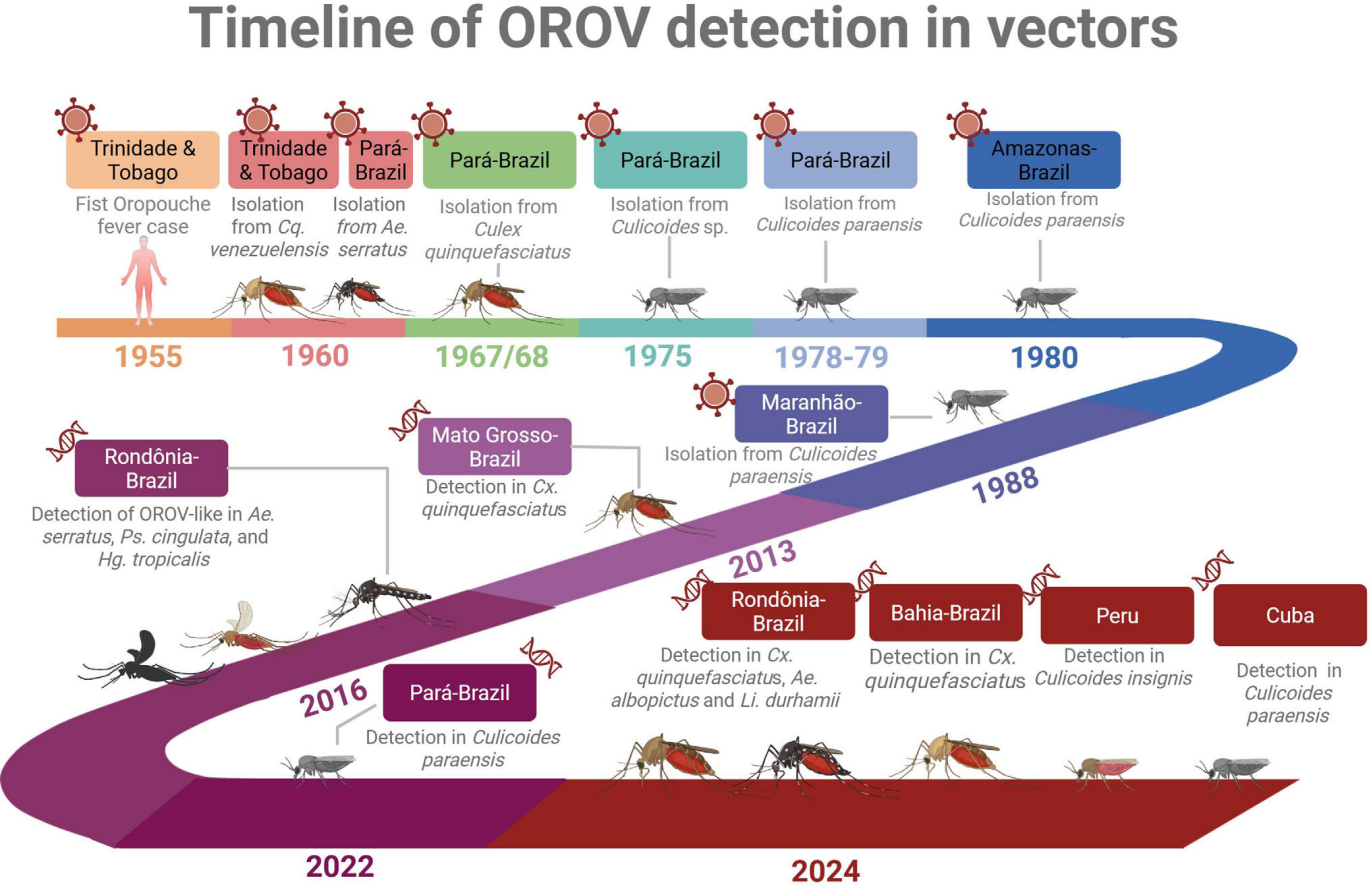
Timeline of Oropouche virus identification in Diptera samples and recording sites, with the techniques used for detection.

As several Oropouche fever cases or outbreaks have been reported in Latin America since 1955,^3^ some studies have attempted to detect the presence of the virus in samples of known anthropophilic mosquitoes collected in urban and wild areas (Fig. 4). Although the first isolations of OROV in hematophagous Diptera occurred from mosquito samples, and a few studies have reported mosquito species naturally infected with OROV in nature, their participation as OROV vectors is not yet confirmed. So far, only a few studies have demonstrated their low or no vector competence to transmit the virus.^21^, ^22^, ^23^, ^24^, ^25^, ^26^, ^27^, ^28^ These results do not provide sufficient evidence to incriminate mosquito species as vectors. It has been observed that OROV replication is restricted by the midgut barrier in mosquitoes.^23^, ^27^

The first detection of OROV in culicidae samples was registered in 1960, from a pool of 177 *Mansonia* (*Coquillettidia*) *venezuelensis* in Trinidad and Tobago.^2^ At that time, no outbreak had been reported in that country. The virus also was isolated in Brazil for the first time in the same year, from the blood of a sloth *Bradypus trydactilus*, captured in São Miguel do Guamá, Pará State, during the construction of the Belém‐Brasília highway. Wild mosquitoes also were collected in the same area, and one isolate was obtained from *Ochlerotatus* (*Aedes*) *serratus*.^29^ In the following year, 1961, OROV was responsible for an epidemic in Belém, Pará state, with ≈11 000 people infected and 15 isolations obtained from human patients. Mosquito collections were conducted during that investigation, and a total of 3262 mosquitoes were sampled, most of which were collected inside the houses of human cases. All mosquito samples were negative for the presence of OROV.^26^ In 1967–1968, three OROV isolations were obtained from *Culex pipiens fatigans* (currently *Cx.. quinquefasciatus* [Say, 1823]) captured in Pará State. In 1971, four OROV isolations were obtained from a sloth captured in Maracanã, Pará State (Travassos da Rosa APA, data unpublished).

In 2013, during a dengue outbreak in Mato Grosso, 387 pools of *Cx. quinquefasciatus* were collected, and eight pools were found to be positive for OROV, following quantitative reverse‐trasncription (qRT)‐PCR analysis.^30^

A retrospective study aimed at detecting OROV and Mayaro virus (MAYV) in an area of intense arbovirus circulation was conducted by Dias *et al*.^31^ The study investigated >22 000 samples from different mosquito species collected between 2016 and 2018 in Mato Grosso State, Brazil, and all samples tested negative for both viruses.

Pereira Silva *et al*.^32^ investigated, in 2019, the potential OROV vectors in the endemic region (Amazon forest). Of the 46 species collected in the study, three were found to be positive for Oropouche‐like viruses: *Haemagogus tropicalis, Ae. serratus* and *Psorophora cingulata*, which were captured in Presidente Figueiredo, a municipality located in the Metropolitan Region of Manaus, Amazonas.

During a 2024 OROV outbreak in Porto Velho city, capital of Rondônia State, 3950 insects were collected and analyzed for the presence of OROV and MAYV. Three mosquito species (*Cx. quinquefasciatus*, *Aedes albopictus* and *Limatus durhamii*) from seven pools tested positive for OROV by qRT‐PCR. That study incriminated *Cx. quinquefasciatus* as the main vector of OROV in that urban transmission cycle.^33^

Da Silva Ferreira *et al*.^34^ studied the natural vertical transmission of arboviruses in mosquitoes in Mato Grosso, the North‐Central region of Brazil, and found two positive pools for OROV, one in *Cx. quinquefasciatus*, from 179 pools analyzed, and one in *Ae. aegypti* from 84 pools. In total, 10 569 mosquitoes were collected and processed, and other arboviruses were detected in different mosquito species. The detection of OROV in mosquito males suggests that vertical transmission in potential vectors is possible, as occurs with other arboviruses in Culicids.

The first attempt to investigate vector competence for OROV transmission in mosquitoes was made by Anderson *et al*.^2^ They evaluated the transmission capacity in four mosquito species: *Aedes scapularis, Ae. serratus, Culex fatigans* and *Psorophora ferox*. The mosquitoes were infected by parenteral inoculation and, after 2 weeks, were allowed to feed on newborn mice. These mouse samples were then used to inoculate infant mice to recover the virus. No transmission was observed using that technique; however, almost 100% of the infected mosquitoes that remained alive tested positive for OROV.

Likewise, Smith and Francy^22^ investigated vector competence in *Ae. albopictus* from Brazil, using MAYV and OROV. Mosquitoes were allowed to feed on infected hamsters and were tested for transmission at 6, 13, and 20 days postinfection (dpi) by refeeding on newborn white mice. The infection rates for both viruses were low, and transmission was only observed for MAYV, with a 50% transmission rate. No transmission was observed for OROV.

The risk of OROV transmission in North America was assessed by investigating the vector competence of two *Culex* species and the biting midge *Culicoides sonorensis*.^24^ *Culex tarsalis* showed a very low infection rate and no dissemination or transmission. *Cx. quinquefasciatus* showed low infection and dissemination rates, and a very low potential for transmission. However, *C. sonorensis* displayed high infection and dissemination rates and limited transmission rates. Based on these results, the authors suggested that *C. sonorensis* could be a putative vector in the USA, given its comparatively higher competence. Using a new strain of OROV isolated in Rome in 2024, Mancuso evaluated the vector competence of *Culex pipens* and *Ae. albopictus* from Italy, and both species exhibited no vector competence.^27^ de Mendonça *et al*.^23^ evaluated the vector competence of *Ae. aegypti*, *Ae. albopictus* and *Cx. quinquefasciatus* for OROV using three different methods: artificial blood meal, intrathoracic injection, and feeding mosquitoes on viremic mice. For both methods using infected blood meals (either via artificial blood feeding or feeding on infected mice), none of the three mosquito species showed vector competence for OROV. However, all three mosquito species were able to replicate OROV when the virus was introduced by intrathoracic injection and subsequently transmitted it to naïve mice. These results suggest that OROV cannot traverse the midgut epithelium or overcome the antiviral defense mechanism in that tissue. Mendonça *et al*. also analyzed the recently isolated OROV strain from Brazil in the most common urban mosquitoes in Brazil, *Ae. aegypti* and *Cx. quinquefasciatus*, and the results demonstrated that the two species are refractory to the virus.^26^

Likewise, using an OROV strain isolated from a patient from Cuba in 2024 and the earliest known strain obtained in 1955 (TRVL9760), Paine *et al*.^25^ assessed the vector competence of four well‐established colonies of mosquito species from the USA: *Ae. albopictus*, *Anopheles quadrimaculatus*, *Cx. quinquefasciatus* and *Cx. pipiens*. The results showed a very low dissemination rate and no transmission, suggesting that all species investigated have no vector competence for both OROV strains.

In Germany, local mosquito populations (*Aedes japonicus*, *Cx pipiens* and *Culex torrentium*) and laboratory colonies (*Ae. albopictus* and *Ae. aegypti*) were tested for the original OROV strain obtained in 1955. Most species have presented low or no infection rate, nor dissemination or transmission. The only exception was *Ae. albopictus* that showed transmission rates ranging from zero to 25.^28^

Although the first isolations were obtained from *Mansonia venezuelensis* (currently *C. venezuelensis* [Theobald, 1912]),^2^ and *Ae. serratus*,^29^ both of which are cited as part of the sylvatic cycle, laboratory confirmation of their vector competence has never been established. Considering that several species also have been found naturally infected with the virus, the main vector involved in the wild cycle remains largely unelucidated. This is an important aspect that deserves detailed investigation, as identifying these species would allow for better prediction of the risk of virus endemicity in areas outside the Amazon region. However, understanding whether the virus can easily adapt to a broad diversity of blood‐sucking arthropod species also is of great importance.

The first association between biting midges and OROV occurred in the Northern region of Brazil, when several outbreaks of Oropouche were reported. Efforts to detect natural OROV infection in hematophagous arthropods have been limited to a few studies, as most investigations focus on human populations. Moreover, the early studies have shown that, unlike other arboviruses, a large number of arthropod samples must be collected to increase the likelihood of detecting OROV in nature.

After several unsuccessful attempts to isolate OROV from mosquito samples during previous outbreaks, it became necessary to investigate other potential vectors. In March 1975, during an investigation of the fifth outbreak of Oropouche fever in the Lower Amazon Mesoregion (Santarém city, Pará state), *Culicoides* spp. samples were collected, as this is a highly abundant group of hematophagous invertebrates. In that study, two OROV isolates were obtained after a total of 15 000 samples (653 pools) of *Culicoides* spp. were inoculated in newborn Swiss mice. The majority of the samples collected were identified as *C. paraensis*. Although mosquitoes also were collected during the investigation, they were not analyzed.^35^ Additionally, in September of the same year, two more pools of *Culicoides* spp. collected in Santarém and Belém cities were found to be positive for OROV isolation (Travassos da Rosa, unpublished data). These findings marked the first time that the possible transmission of a virus of medical importance in humans by Culicoides was identified.

Owing to the difficulty in obtaining the virus from vector samples, several attempts were made to characterize OROV vectors. Therefore, epidemiological investigations were conducted in conjunction with entomological investigations. Roberts *et al* ^36^ summarized the relationships between these studies during the 1975 epidemic in Pará. These studies gave preference to capturing vectors using human bait in order to attract the most anthropophilic species. The idea was to correlate the spatial distribution of disease cases with the local abundance of hematophagous insects in the affected areas at the time of the outbreaks. More than 3000 insects were collected, and a positive correlation between *Culicoides* and *Cx. quinquefasciatus* with Oropouche fever cases was observed; however, this correlation was strongest for *C. paraensis*. They concluded that this species was the main urban vector of OROV.

In 1978, in a small village near Belém, Pará State, just after the OROV outbreak, LeDuc *et al*.^37^ conducted a house‐to‐house survey. Human samples were collected, and several species of hematophagous arthropods were collected and investigated. Only unfed insects were analyzed. A total of 393 pools were tested. Three species of *Aedes*, four species of *Culex*, two species of *Psorophora*, *Trichoprosopon digitatum* and *Anopheles albitarsis* constituted 50 pools, and 60 453 *C. paraensis* constituted 343 pools. Only four pools of *C. paraensis* tested positive, and the virus was isolated.

In 1979, an Oropouche fever outbreak was reported in the eastern region of Pará state. A total of 21 174 *Culicoides* specimens were collected during the outbreak and grouped into 671 pools. One pool collected in Belém, the capital of Pará state, tested positive for OROV, and a strain was successfully isolated. No virus was obtained from the 4740 *Cx. quinquefasciatus* specimens collected during the investigation, nor in any of the other culicids collected in the study.^38^, ^39^

During the outbreak of Oropouche fever in Amazonas State in 1980, an entomological investigation was conducted in sites where acute cases were occurring, including a hospital, a school and residential houses. Only two species were collected: *C. paraensis* and the southern house mosquito, *Cx. quinquefasciatus*. The number of biting midges collected (5848) was five‐fold more numerous than mosquitoes (1034). Only one pool—comprising 50 *Culicoides* specimens collected at the hospital—resulted in OROV isolation.^40^

Dixon *et al*.^41^ while investigating an Oropouche fever outbreak in the city of Santarém, Pará State, concluded that, despite not having isolated the virus from biting midges, *C. paraensis* was probably the primary vector of the virus. This conclusion was based on the observation that the incidence rate of the disease was significantly higher in women than in men in that city, which was associated with the different host‐feeding patterns of the two species: *C. paraensis* and *Cx. quinquefasciatus*.

In 1988, during an OROV outbreak in Goiás (Midwest of Brazil) and Maranhão (Northeast of Brazil), >3600 *C. paraensis* and 1970 *Cx. quinquefasciatus* were collected from houses where human cases were reported, along with other dipteran species. However, only one sample of *C. paraensis* collected in Porto Franco city, Maranhão, resulted in virus isolation, suggesting that the infection rate in vectors is very low.^42^

By contrast, at the end of 1994, a large Oropouche fever outbreak was reported in Serra Pelada, Pará State, with an attack rate of ≈83%. Among the tested samples, 82.8% were positive, yet all *Culicoides* and mosquito samples tested negative for OROV. In total, 2400 biting midges and 1036 mosquitoes (mostly *Cx. quinquefasciatus*) were processed and inoculated intracerebrally in newborn Swiss mice.^43^

Likewise, in Rondônia, another state located in the Brazilian Amazon region, Culicoides specimens were collected during an interepidemic period between 2019 and 2020, and analyzed for the OROV detection. In total, 7315 *C. paraensis* were collected, and all tested negative. The study also confirmed that this species exhibits diurnal activity and is more abundant during the rainy season.^44^

In a recent entomological survey during the 2024 outbreak in the Peruvian Amazon, two positive pools of *Culicoides insignis* were reported. A total of 61 mosquito pools were analyzed, = all testing negative. *C. insignis* was the most abundant species collected in the Ucayali region, representing 96.7% of all Culicoides collected in that area, and this species was considered a possible new vector for OROV.^45^

Notably, although the 2023–2025 Oropouche fever outbreaks in Brazil affected numerous extra‐Amazonian states, no official or peer‐reviewed studies to date have reported entomological detection of OROV in *Culicoides* or mosquito specimens in these areas. Surveillance efforts are documented epidemiologically and reinforced by alerts from PAHO/WHO or Brazilian Ministry of Health authorities, but they do not provide official laboratory data confirming vector positivity outside the Amazon region. However, during a webinar held on 13 March 2025, by RELDA and RELEVA, preliminary unpublished data from the Evandro Chagas Institute (Brazilian Arboviruses National Reference Laboratory) were presented, revealing multiple *C. paraensis* specimens positive for OROV collected during the outbreak in Espírito Santo State, as well as an engorged *Cx. quinquefasciatus* specimen captured in the state of Bahia.^46^

In Cuba, during the 2024 OROV outbreak, *C. paraensis* samples were collected in areas where cases had been reported. The authors emphasized that the species were collected exclusively through human landing catches (HLC), but not using any type of traps.^47^

In order to demonstrate the vector competence of *C. paraensis*, studies were conducted using samples collected directly in the field, as maintaining these insects under laboratory conditions proved difficult. *Culicoides* were collected in Belém and fed on infected hamsters previously inoculated with OROV at different concentrations. Afterwards, these potentially infected midges were fed again on naïve hamsters at several time points postinfective blood meal. The biological transmission of the virus was successfully demonstrated; they found a higher transmission rate (83%) for *Culicoides* infected with a lower titer infectious blood meal, compared to *Culicoides* infected with a higher viral dose (25%).^18^ Those studies also demonstrated that hamsters can be infected by the bite of a single *Culicoides*. In the following year, the transmission of OROV from man to hamsters through *C. paraensis* bites also was demonstrated. Between 20 and 100 Culicoides were placed on the infected patient's hands for 1 h, and after 5 days, these midges were fed on hamsters, which were monitored daily. Sick animals were used to detect the presence of OROV by complement fixation using brain and liver tissues. Animals with no clinical signs were investigated for the presence of OROV antibodies after 3 weeks of exposure. They found a transmission rate ranging from 40% to 50%. This study provided conclusive evidence of Culicoides as an OROV vector for the first time. The authors suggested that the extrinsic incubation period is only 5 days.^48^ A recent study compared the vector competence of *C. sonorensis* for two OROV lineages: the historical one obtained in 1960, and the one recently isolated from the 2024 outbreak in Cuba. The authors observed a shorter extrinsic incubation period and higher rates of infection, dissemination and transmission for the 2024 lineage. Additionally, the 2024 isolate replicated to higher viral titers in midge cells compared to the historic isolate.^49^

## 4 CHALLENGES IN ENTOMOLOGICAL SURVEILLANCE AND VECTOR CONFIRMATION OF OROV

**Figure 5:**
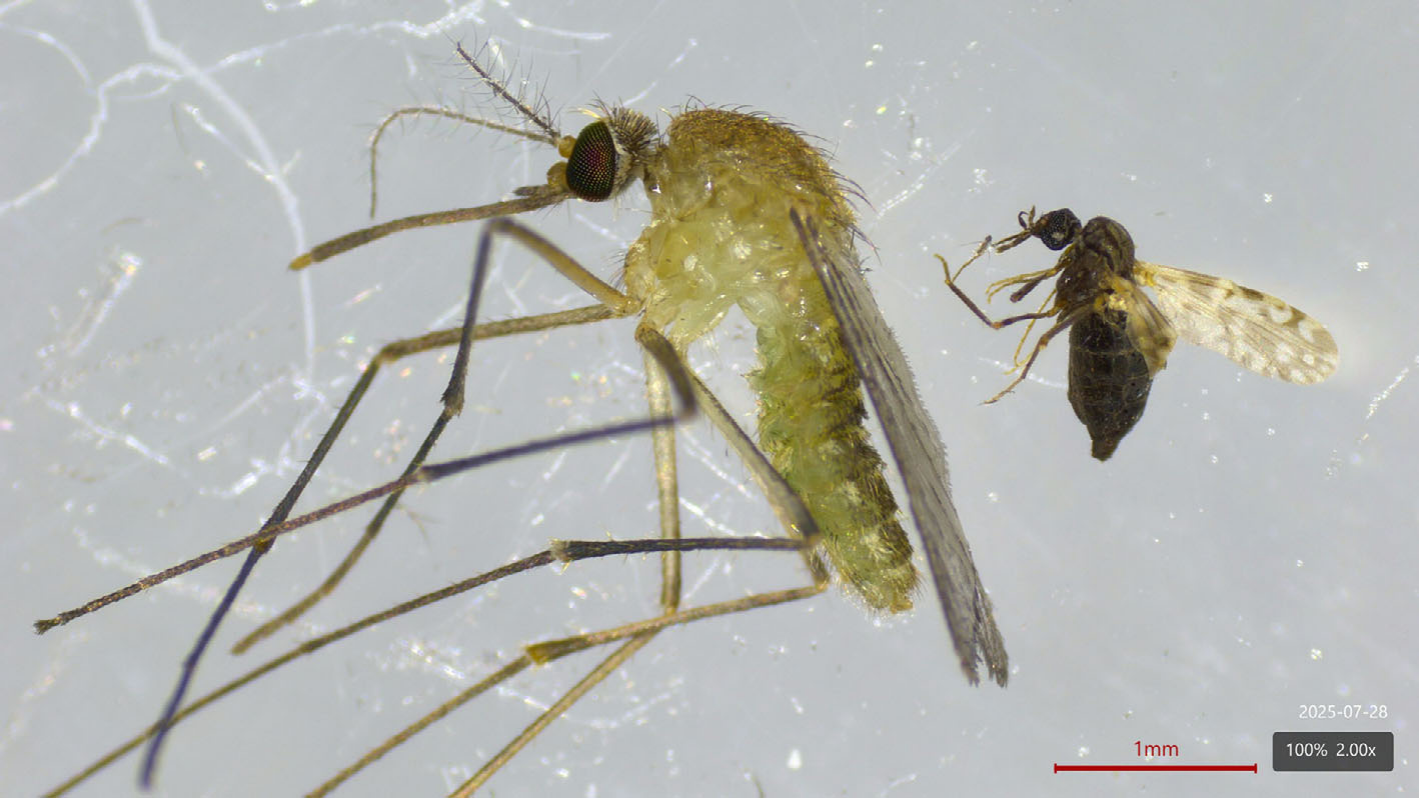
Size difference between female specimens of *Culex quinquefasciatus* and *Culicoides* spp.

Several factors may explain the difficulty in obtaining positive samples from vectors:

- The high abundance of *Culicoides* populations in the natural environment makes it challenging to detect naturally infected individuals, because human infection occurs sporadically, incidentally, and typically outdoors, where the resting sites of biting midges are extremely difficult to locate and sample.
- Furthermore, the viremic period for patients infected with OROV is not well‐characterized, but probably follows the same pattern for other arboviruses, which is relatively short (≈5 days), therefore reducing the chances of infecting new vectors.
- The duration of infection in Culicoides after virus acquisition is unknown and requires further investigation. In most cases, by the time a laboratory diagnosis of Oropouche fever is confirmed, several weeks have passed since symptom onset—potentially exceeding the lifespan of infected insects—thus narrowing or eliminating the window of opportunity for field vector collection.
- Considering the size of the biting midges (Fig. 5) and the low titer typically observed for OROV in field samples, the viral load is probably too low to be detected by qRT‐PCR. It is possible that current RNA extraction methods need to be re‐evaluated and optimized for such low‐input samples. Otherwise, pools with >50 to 100 individuals are necessary to increase the chance of detecting positive samples.

*Culex quinquefasciatus* exhibits endophilic behavior in the Neotropical region, typically resting indoors. This increases the chances of contact with OROV‐infected individuals, especially those who are bedridden, allowing the mosquito to feed throughout the entire viremic period. Additionally, *Cx. quinquefasciatus* is easier to collect in indoor environments, facilitating entomological investigations. Owing to its larger body size compared to biting midges (Fig. 5), the mosquito ingests a greater volume of blood, which may result in a higher viral load. This increases the likelihood of OROV detection by molecular techniques or viral isolation in cell culture, as compared to *Culicoides*.

It is therefore essential to distinguish a true biological vector from an insect that has merely fed on a viremic host. This distinction is particularly challenging for biting midges. Even when multiple positive field samples are detected, performing vector competence studies remains difficult owing to the absence of established *Culicoides* colonies in insectaries across the Neotropical region.

In summary, data in the literature are scarce but suggest that *Culicoides* are the main vectors. If *Cx. quinquefasciatus* were significantly involved in the transmission, one would expect an explosion of cases in the capital cities of other regions in Brazil outside the Amazon forest. In the Northeast region, for instance, all Oropouche fever cases from the 2023–2025 outbreak are concentrated in the Atlantic Rain Forest, where the ecosystem is more favorable to biting midges, with fruit trees such as banana and cocoa commonly found. There is a great diversity of *Culicoides* in this region, and identifying the actual vector species will be a major challenge. By contrast, *Cx. quinquefasciatus* has been found in high densities in urban centers, particularly in areas characterized by poor socio‐environmental conditions.^50^ This species also is widely distributed in other biomes such as the caatinga dry forest and Brazilian savanna.^51^ According to the SES‐PE *Boletim Epidemiológico de Arboviroses n° 14*,^52^ 179 confirmed cases of Oropouche fever were reported in Pernambuco State, concentrated in the Zona da Mata (Atlantic Rain Forest) and Agreste regions, and just a few cases in the urban area in Recife city, the state capital. These ecological patterns further suggest that *Cx. quinquefasciatus* may not play a central role in the current transmission dynamics of OROV. Taken together, the analyses presented here are consistent with the conclusions of Pinheiro *et al*.,^53^ which incriminate *C. paraensis* as the primary vector of OROV and exclude the possibility that *Cx. quiquefasciatus* is the epidemic vector.

Regarding the sylvatic cycle, *C. venezuelensis* and *Ae. serratus* have been positive to OROV detection; however, there are no vector competence studies published for wild mosquito species, and this remains an important gap to be addressed in future studies. Additionally, the environmental factors that determine the abundance of these species must be investigated, along with the diversity of vertebrate hosts that serve as a blood source for *Culicoides* females and that could explain the maintenance of a sylvatic OROV cycle in the region. Vieira *et al*.^54^ demonstrated that mosquito bloodmeals can be highly informative in characterizing this diversity of hosts. The good news is that it is easy to collect engorged biting midge females using light traps; however, not all species are attracted to these traps.

## 5 PERSPECTIVES AND RESEARCH AGENDA

Whole‐genomic sequencing of several arbovirus strains has experienced remarkable growth in recent years, driven by advances in next‐generation sequencing technologies. These data have become increasingly relevant to developing vaccines, drugs and diagnostic tests, and finally, to understanding the epidemiological scenarios of the arboviruses. However, much less attention has been given to studies of the biology and ecology of vectors, particularly the components involved in vectorial capacity, which are important factors in the complex pathogen–vector–host relationship. Without understanding which vector species are involved in virus transmission, their ecology and population diversity, and their environmental dependence, it is premature to attribute the determinants of re‐emergence solely to viral evolution.

Therefore, investigations that focus on the characterization of vector species, shifts in the abundance and distribution of primary vectors as a consequence of climate change, and the impact on the ecology of these species must be prioritized. Furthermore, whole‐genome sequencing of *Culicoides* species needs to be conducted as soon as possible. These efforts will provide essential data to support new research, inform entomological surveillance, and guide the design of effective vector control strategies aimed at reducing the burden of the disease.

Considering the points discussed above, and the ongoing spread of OROV, which continues to cause a high number of cases in Brazil and other Latin American countries, it is imperative to establish a research agenda aimed at filling the existing knowledge gaps about this arbovirus and its vectors. Priority should be given to entomological investigations that explore the diversity, abundance and seasonality of vector species in outbreak areas, as well as their ecological and behavioral characteristics. Particular attention should be paid to assessing the vector competence of wild mosquito species and identifying their bloodmeal sources, which are key to clarifying the dynamics of the sylvatic transmission cycle. In parallel, it is necessary to develop standardized and comparable surveillance protocols to improve the accuracy of outbreak investigations. Additionally, operational research should evaluate the effectiveness of available vector control tools and repellents for biting midges, which remain largely understudied. Finally, public health strategies must include targeted education efforts to reduce exposure risk, especially in vulnerable communities living in high‐transmission areas.

## 6 RESEARCH AGENDA ON OROPOUCHE VIRUS VECTORS

- To describe the diversity of the species occurring in the outbreak areas.
- To characterize the abundance and seasonality of the species.
- To assess the vector competence of wild mosquito species, including identifying their blood source, to characterize the wild cycle of OROV transmission.
- To establish robust protocols for entomological surveillance during outbreaks that are comparable across studies.
- Whole‐genome sequencing of *Culicoides* species.
- To establish new *Culicoides* colonies in insectaries or ACL‐2 (Arthropod Containment Level 2) to enable standardized studies.
- To test products that can be used for vector control of biting midges.
- To test different compounds that can be used as repellents.
- Develop health education strategies to reduce the risk of exposure for the most vulnerable communities.

## CONFLICT OF INTEREST

The authors declare no competing interests.

## AUTHOR CONTRIBUTIONS

CFJA made the literature search, study design, data collection, data analysis, data interpretation, and writing. DBAM made the literature search, Figures design, data analysis, data interpretation, and writing. BLSN and JPNN made data interpretation and writing. All authors reviewed the final version of the manuscript.

## Data Availability

Data sharing not applicable to this article as no datasets were generated or analysed during the current study.
